# Small-area methods for investigation of environment and health

**DOI:** 10.1093/ije/dyaa006

**Published:** 2020-03-17

**Authors:** Frédéric B Piel, Daniela Fecht, Susan Hodgson, Marta Blangiardo, M Toledano, A L Hansell, Paul Elliott

**Affiliations:** d1 UK Small Area Health Statistics Unit, Department of Epidemiology & Biostatistics, School of Public Health, Imperial College London, London, UK; d2 MRC-PHE Centre for Environment & Health, Department of Epidemiology & Biostatistics, School of Public Health, Imperial College London, London, UK; d3 National Institute for Health Research Health Protection Research Unit (NIHR HPRU) in Health Impact of Environmental Hazards, Imperial College London, UK; d4 Centre for Environmental Health and Sustainability, Medical School, University of Leicester, Leicester, UK

## Abstract

Small-area studies offer a powerful epidemiological approach to study disease patterns at the population level and assess health risks posed by environmental pollutants. They involve a public health investigation on a geographical scale (e.g. neighbourhood) with overlay of health, environmental, demographic and potential confounder data. Recent methodological advances, including Bayesian approaches, combined with fast-growing computational capabilities, permit more informative analyses than previously possible, including the incorporation of data at different scales, from satellites to individual-level survey information. Better data availability has widened the scope and utility of small-area studies, but has also led to greater complexity, including choice of optimal study area size and extent, duration of study periods, range of covariates and confounders to be considered and dealing with uncertainty. The availability of data from large, well-phenotyped cohorts such as UK Biobank enables the use of mixed-level study designs and the triangulation of evidence on environmental risks from small-area and individual-level studies, therefore improving causal inference, including use of linked biomarker and -omics data. As a result, there are now improved opportunities to investigate the impacts of environmental risk factors on human health, particularly for the surveillance and prevention of non-communicable diseases.

**Key Messages**

- Small-area methods have been extensively used in public health practice in the UK and other high-income countries, and could be used in similar ways in low- and middle-income countries as relevant data become available.
- Rapid data linkage is essential to make the most of environmental, health, demographic and confounder data available from a wide set of geographies for surveillance, investigations of environmental health risks and the prevention of non-communicable disease.
- New methodological advances in statistical methods, including Bayesian approaches and use of mixed-level designs, together with advances in computational capacity, allow the simultaneous investigation of multiple health outcomes and multiple environmental exposures while quantifying uncertainty throughout studies.

## Introduction

A range of environmental exposures may impact human health, but our level of understanding and awareness of these links is highly variable. Although there has been a focus on the health effects of outdoor air pollution,^1–3^ climate change^4–6^ and ionizing radiation (e.g. nuclear power plant accident or waste),^7^^,^^8^ possible health risks associated with other widespread exposures, such as noise pollution,^9^^,^^10^ non-ionizing radiation from mobile phones and other electro-magnetic sources,^11^^,^^12^ and microplastics^13^ are emerging. The rapidly increasing volume of available routine health and environmental data offers new opportunities to better understand and assess risks for human health and to guide public health policies. The smoking bans enforced throughout Europe in the 2000s and the recent sugar tax implemented in Mexico and the UK highlight the potential for rapid impact of such public health policies.^14^^,^^15^ Nevertheless, the inability to date to reduce air pollution to legal or recommended levels in London and other large cities reflects some of the challenges involved in translating scientific evidence into policies and their implementation.^16^

Studies assessing health risks from environmental factors ideally need to: (i) involve large populations to gain sufficient statistical power to investigate relatively rare health events, and to detect the effects of low to very low levels of pollutant exposure; and (ii) be comprehensive in terms of geographical and population coverage, as risks vary in space and time, as well as by age, sex, sociodemographic status, and other possible confounders. Fulfilling these criteria for individual-level epidemiological studies across entire populations or an ad hoc subset of the population (e.g. within an exposed area) often remains challenging. Although the size of cohort studies collecting in-depth individual-level data has considerably increased in recent years (e.g. 500 000 participants in UK Biobank),^17^ it is still beyond scope to collect such data across entire populations. Small-area studies offer an alternative study design based on spatial epidemiological analyses of individual or aggregate data at the neighbourhood scale (e.g. a few blocks/streets, lowest census geography).^18^ Populations within small areas tend to be more homogeneous than in larger areas, providing a differential between the socioeconomic and environmental characteristics of areas studied that may aid detecting relationships between these variables and health data.^19^

In small-area studies, individual exposure is often assigned based on one location—residence, workplace or school. Such assignment then makes it possible to map disease risks and pollutant concentrations, and to investigate health risks associated with local exposures at the population level. The small-area study design is particularly useful to: (i) approximate individual-level risks when individual-level data are either limited or unavailable^20^; (ii) investigate risks to health from sources of environmental pollution^21^; (iii) detect high-risk areas and plan appropriate interventions^22^; and (iv) conduct initial investigations of reported disease clusters.^23^ Small-area studies often rely heavily on the availability of health, environmental, demographic and confounder data across entire populations or large subsets.^24^ Examples from over 30 years of experience in conducting small-area environment-health analyses by the UK Small Area Health Statistics Unit, SAHSU [www.sahsu.org],^25^ include studies of waste disposal,^26^^,^^27^ temperature extremes,^4^^,^^28^ air and noise pollution,^9^^,^^29–33^ chlorination by-products in the water supply^34^^,^^35^ and electromagnetic fields from overhead power lines and mobile phone masts.^11^^,^^36^

Better data availability can widen the scope and utility of small-area studies.^37^ It can also lead to greater complexity, including the choice of the optimal study area size and extent, the duration of study periods and the range of covariates and confounders to be considered. Here, we discuss these key methodological choices in light of recent methodological advances, including Bayesian approaches,^38–40^ which help to link and process large volumes of available data. Together with a discussion of future challenges going forward, we aim to summarize the basis for rigorous analyses of environment and health risks using the small-area approach.

## Methodological choices

When conducting a small-area study, a series of methodological choices need to be made which may influence the identification and interpretation of an environmental health risk. These include identification of the available data sources, choice of geographical scale and study duration, and application of appropriate statistical methods for the analysis.

### Data sources

**Figure 1:**
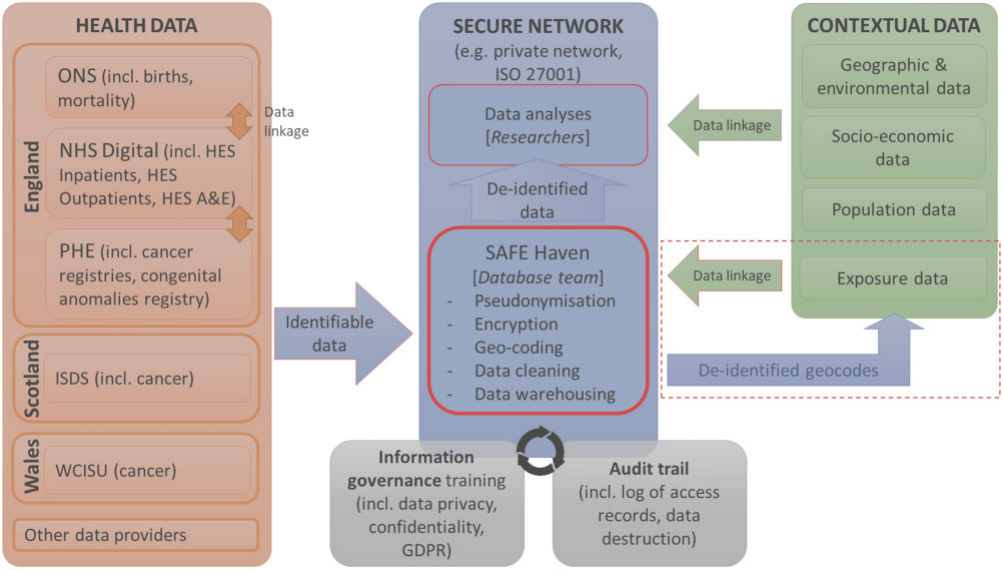
Schematic of a secure data network such as used by the UK Small Area Health Statistics Unit for small-area studies. ONS, Office National for Statistics; PHE, Public Health England; HES, Health Episode Statistics; A&E, accident and emergency; ISDS, Information Services Division Scotland; WCISU, Welsh Cancer Intelligence & Surveillance Unit; GDPR, General Data Protection Regulation; ISO, International Organization for Standardization.

Small-area studies typically involve a range of health, population and environmental data across standard geographies (Figure 1). For example, to study reproductive effects associated with a risk factor or local pollutant (e.g. incinerator proximity), individual data on birthweight, stillbirth and/or congenital anomalies, population data on births, and information on potential confounding by socioeconomic variables and ethnicity based on census data are required, alongside measured or modelled exposure data.^41^

#### Health data

The volume and accessibility of health records have dramatically increased in the past 2-3 decades. In England, on average over 100 million individual records from outpatient, maternity, adult critical care, and accident and emergency services across all NHS hospitals are added every year to the Health Episode Statistics, HES [http://content.digital.nhs.uk/hes] database. SAHSU, which holds and maintains databases of health and geographical data, social confounding factors and environmental exposures required to conduct small-area health studies, holds more than 600 million individual health records in a secure system (Figure 1).

**Figure 2:**
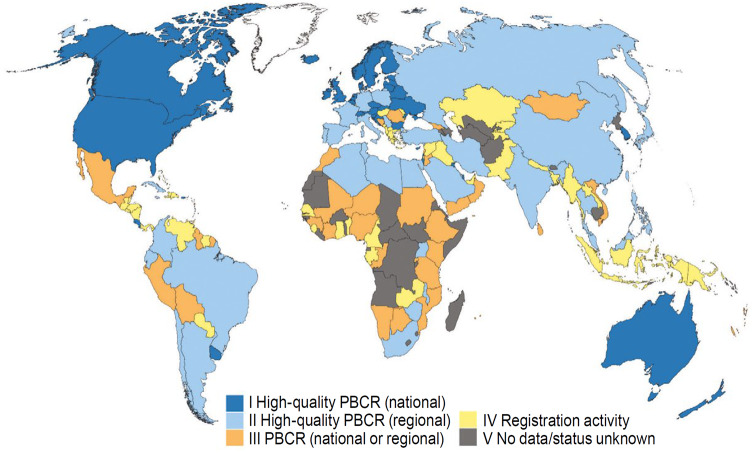
Quality of population-based cancer registries per country in 2013. PBCR, population-based cancer registry. Reproduced with permission from Bray *et al*^42^.

The coverage of routinely collected health data varies substantially between countries. NHS data in England offer near universal coverage of births, cancer, hospital admissions and mortality which allows investigation of spatio-temporal health patterns in neighbourhoods and local areas for any part of the country. Detailed records are entered for over 1 million patient events every 36 h within the NHS. Nevertheless, there are very limited routine health data being collected, particularly in digital format, in many rural areas across low- and middle-income countries. These disparities appear, for example, in the quality of population-based cancer registries (Figure 2).

Even when good coverage is available, the completeness and quality of datasets need to be assessed (Supplementary Material 1, available as Supplementary data at *IJE* online). Most health databases typically miss a subset of the population. Although the impact of random gaps may be limited, the mis-representation of specific sub-groups (e.g. homeless, migrants, refugees and asylum seekers)^43^ needs to be carefully considered. Patients going to private practices in England—about 11% of the UK population have a private health insurance in addition to free access to the NHS^44^—are not recorded in NHS databases, which may lead to an underestimation of some health conditions, particularly in the most affluent sub-groups.^45^ Age, sex, ethnicity and sociodemographic status influence the prevalence of opt-outs in the NHS, whereby patients can choose not to share their health record beyond purposes relating to their direct clinical care.^46^ Spatial visualization can help identify data quality or completeness issues. By mapping data on births at small-area level in London, Ghosh *et al.* identified missing data from local hospitals in south-east London.^47^ Finally, the quality and completeness of a dataset can vary over time due to issues such as staff training, data collection methods and changes in disease classification (e.g. International Classification of Diseases codes).

Because the number of cases for a given condition at the small-area level is often limited, ensuring the highest quality of health data is essential for correct interpretation and identification of potential risk factors. This is particularly relevant when studying rare conditions such as congenital anomalies.^48^ Duplicate cases in the database can give rise to spurious ‘clusters’, and gaps in the data may be detected as ‘holes’ in a mapping surface.

#### Population data

Census data are often used to provide background population counts of individuals at risk, as well as sociodemographic covariates (e.g. age, sex and deprivation) and potential confounders (e.g. smoking). Intercensal estimates, sometimes provided with precision measures as in the American Community Survey,^49^ provide annual population and demographic data at various sub-national geographies (Table 1). In countries using decennial censuses, inaccuracies in denominator information tend to be higher in intercensal years.^50^ Inaccurate estimates can change the patterns observed when data are mapped and can complicate map comparisons, especially for areas with small populations. Small-area studies require spatially and temporally detailed population data as denominators for calculating rates or risks. The quality of population counts is therefore vital for any health analysis and health surveillance.

**Table 1.** Hierarchical administrative units used in England, illustrating the inverse relationship between the size of a unit and its population

Geographical unit	Number of units	Population per unit
(England, 2011 Census)		
Country	1	53 107 000
Local authority	324	25 000–1 000 000
MSOA	6791	5000–15 000
LSOA	32 844	1000–3000
Census output area	171 372	100–625
Postcode	1 745 912	43
Address/household	22 000 000	2.4
Individual	53 107 000	1

Based on information available from the Office for National Statistics (ONS) [www.ons.gov.uk/methodology/geography/ukgeographies/censusgeography].

MSOA, middle layer super output area; LSOA, lower layer super output area.

#### Environmental data

Exposure assignment at the population level relies mostly on exposure proxies. A traditional approach in small-area studies is distance-based analyses, usually between the place of residence and the source of a pollutant (e.g. distance to road or to industrial chimney stack).^51^^,^^52^ Populations at risk can be stratified by distance from the source of emission or contamination.^53^ A categorical approach—for example near, intermediate and distant—may help to assess the presence or absence of a risk to health. Whereas distance to source may be based on either the small-area geometric or population-weighted centroid, more precise distances can be calculated when individual-level data (i.e. the residential postcode or address) are available. This is particularly important if the spatial distribution of exposure drops off rapidly with distance, e.g. air pollution from a road source or electromagnetic fields from a power line.^54^

Small-area studies are increasingly using sophisticated modelling techniques to provide proxy estimates of individual pollution exposures for place of residence, ideally with validation data representative of the areas involved, or multiple locations (e.g. dynamic mobile health geography).^55^ London Air [https://www.londonair.org.uk/] provides air pollution estimates for 20-metre grids, which can be linked to individual addresses and then be used in epidemiological studies.^56^^,^^57^ Dispersion patterns are well known for some pollutants, but simple models might be more appropriate for those where specific exposure pathways are less clear.^58^

Likewise modelling of exposures from point (e.g. atmospheric dispersion model system emissions modelling for incinerators)^27^ or line sources (e.g. 3D modelling using data on building heights for air and noise pollution along streets)^32^ can be used to assign such exposures to individual postcode or address.

#### Confounder data

**Figure 3:**
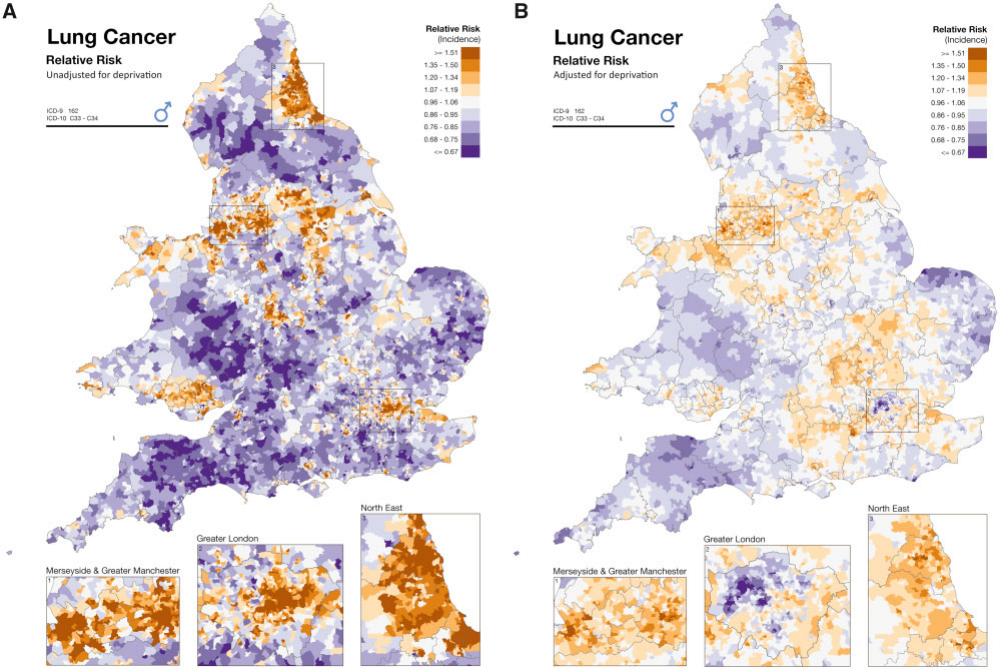
Map of the smoothed relative risk of male lung cancer unadjusted (A) and adjusted (B) for deprivation, using the Carstairs Index, at ward level in England. Data have been adjusted for age. Not adjusting for deprivation increases the observed variability of the disease, whereas adjustment shows many more areas of average risk (white) and fewer areas of very high or very low risk (dark orange or dark purple). In the case of lung cancer, adjustment for deprivation will partly adjust for individual smoking effects (as smoking rates are higher in more deprived areas)—smoking being by far the strongest risk factor for lung cancer. Reproduced with permission: Hansell AL, Beale LA, Gosh RE, Fortunato L, Fecht D, Jarup L, Elliott P . *Environment and Health Atlas for England and Wales*. 2014. www.envhealthatlas.co.uk.

Like any epidemiological study, small-area studies are susceptible to confounding, which can result in spurious exposure–disease associations. As a special type of ecological study, they are also prone to the ecological fallacy,^20^ although the small-area design attempts to minimize this by using small geographies that provide a closer estimation to individual-level risks. Diseases and outcomes usually vary by age and sex, which can be addressed by standardization.^59^ Differences in the socioeconomic status of areas is a major potential source of bias in small-area epidemiological studies (Figure 3), as socioeconomic factors are strongly associated with disease occurrence, and deprived areas tend to have higher levels of environmental exposures (e.g. industry and pollution), whereas affluent areas are usually greener: so-called ‘environmental justice’.^60^^,^^61^ Multiple indices of deprivation have been used to capture socioeconomic differences, for example in the UK: the Townsend Index, the Carstairs Index and the Index of Multiple Deprivation (IMD).^62–64^ The complexity of the indices has increased over time to capture different components of deprivation. The IMD 2019, for example, incorporates data on a range of dimensions including income, employment, health and disability, education, crime, barriers to housing and services, and living environment. The IMD 2019 ranks the 32 844 Lower Layer Super Output Area across England from least deprived to most deprived, although the inclusion of health variables in the IMD complicates its interpretation in health studies. As a result, although it represents only a small contributor to the overall index, it is preferable to remove this component when using the IMD for health study analyses. Choosing the most appropriate index depends on availability for a specific area or country, as well as the ability to compare data across different time periods or areas.^64^ There can also be institutional preferences so that, for instance, the Carstairs Index is largely used in the Scottish NHS and the IMD is mostly used in local government in England.

Housing, wealth, diet, lifestyle exposures (e.g. smoking) and access to medical care are all associated with the health of the population. Smoking behaviour is a key potential confounder. In England, direct information on smoking by area is not readily available, although smoking is strongly associated with deprivation, so that at least to some extent it is being controlled for by use of deprivation indices.^65^ In addition, lung cancer mortality has been used as an indirect indicator of community cumulative smoking exposure.^66–68^

Finally, ethnicity may need to be considered, as disease risks may vary between populations of different ethnicities, and ethnic minority populations may tend to live in specific areas of a city, region or country. This is well illustrated in small-area studies on diabetes.^69^ In the SAHSU study of cardiovascular risks related to aircraft noise near Heathrow airport,^9^ adjusting for ethnicity was important as there is a large South Asian community living in West London near the airport, and South Asians are known to be at higher risk of cardiovascular disease independently of aircraft noise exposure.

#### Biomarker data

There is a growing number of studies (e.g. UK Biobank) collecting data on biomarkers, which may provide a valuable, person-specific measure of dose. Biomarkers can be extracted from biological samples such as saliva, blood or urine and may offer a biological measure of current or historical exposure to a pollutant, or a biological indicator of presence of disease. They may allow detection of biological changes due to environmental exposures which may not have been previously detected. Although to date biomarkers have rarely been used in small-area studies, they could help evaluate findings in epidemiological analyses.^70^^,^^71^

#### Linkage between datasets

**Figure 4:**
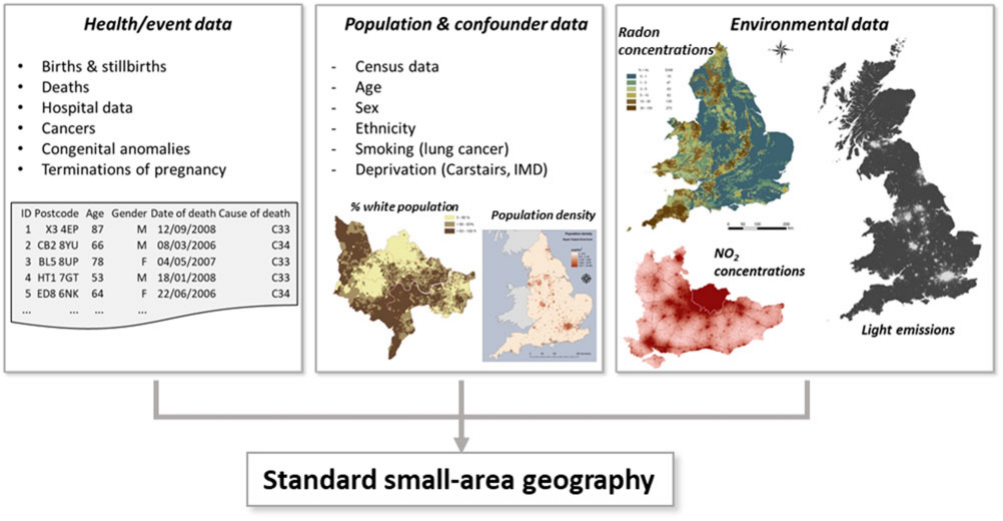
Schematic of the data linkage between health, sociodemographic and environmental data to standard geographies. IMD, Index of multiple deprivation.

Linkage between health datasets can provide valuable information for long-term follow up of specific individuals. For example, combined data on hospital admissions from the Hospital Episode Statistics and mortality from the Office of National Statistics datasets provided by NHS Digital offer additional valuable information, such as the cause of deaths and data on deaths which occurred outside hospital settings. Developing standard geographies across environmental, health and sociodemographic data is essential to conduct small-area studies of environment and health associations (Figure 4).

The borders of administrative units often change over time, so this can be particularly challenging for studies of long-term health impacts. When exposures vary over short distances, the linkage of environmental and health data should be conducted at the individual level for optimum accuracy. For example, the Avon Longitudinal Study of Parents and Children links participants to residential address, health, political and administrative geographies across their life course, as well as to neighbourhood data including deprivation and environmental exposures.^72^

### Selection of study area

Defining the study area, and a reference area if appropriate, is a critical step. *Post hoc* definition of the study area may lead to bias if the boundaries are drawn tightly around an area of disease excess—the so-called Texas sharpshooter fallacy.^73^ The study area may range from a small region to a whole country or group of countries. The reference area is usually a larger geographical area used to compare the health risks of the study area population with those of the reference area population, for example the surrounding region or the national population. Various standardization methods, including direct and indirect, may be used to allow for comparisons between areas.^74^

**Figure 5:**
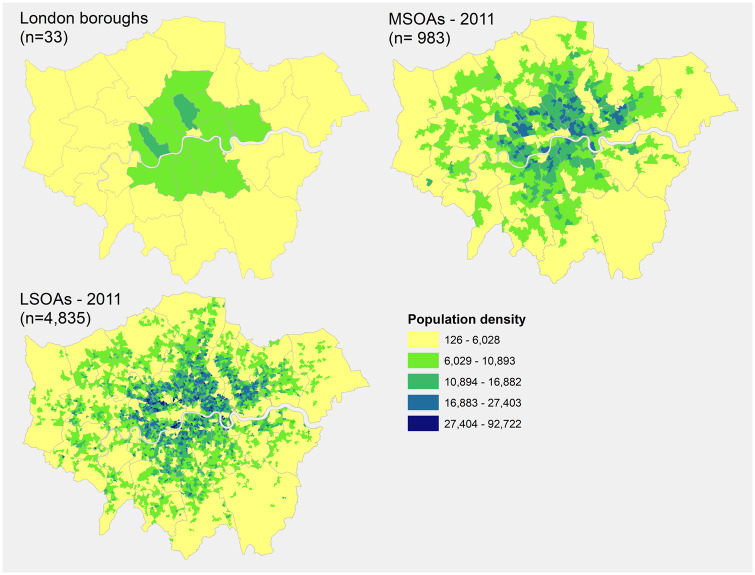
Population density (inhabitants per square kilometre) in the Greater London area. The three maps depict the same data at three different scales: boroughs (London average: ±249 000 inhabitants), middle layer super output areas (MSOA, England average: ±7000 inhabitants) and lower layer super output areas (LSOA, England average: ±1500 inhabitants). The borough-level map masks most of the local variability, and outliers or unstable measurements are more likely to be found at the lower layer super output area level. Contains National Statistics data © Crown copyright and database right [2011]. Data obtained from the London DataStore [https://data.london.gov.uk/dataset/super-output-area-population-lsoa-msoa-london].

Mapping a dataset at different scales can lead to different maps that emphasize different features of the data (Figure 5). Problems can arise from the imposition of artificial units of spatial reporting (e.g. administrative units) on continuous geographical phenomena, resulting in the generation of artificial spatial patterns. This is commonly referred to as the modifiable areal unit problem.^75^ The choice of the most suitable level is driven by: (i) the availability of data—aggregated data are increasingly becoming freely accessible online, but data at small-area level may contain potentially identifiable personal information (e.g. small numbers of a rare disease) and therefore restrictions on the unit of analysis apply in relation to both the numerator and the denominator in accordance with the specific policies of the data providers involved; (ii) the frequency of the event studied—the study of rare events such as congenital anomalies at a very fine geographical scale will inevitably lead to small numbers and an over-representation of zero counts; and (iii) the precision of risk estimates—power calculations will provide information about the sample size needed to detect a defined level of risk: the lower the excess risk to be detected, the larger the study population and years of observation will be needed to detect that risk.^76^

Once the appropriate scale is identified, careful consideration needs to be paid to any temporal changes in the geographical units. For example, postcodes (*n* = 1 765 422 in late 2019) in the UK are issued, re-allocated or deleted on a regular basis, with thousands of postcodes added and deleted every year [https://www.bph-postcodes.co.uk/guidetopc.cgi]. Ignoring these changes can lead to gaps or inaccuracies in data which may influence the study results, especially those linked to a specific local area. The NHS Postcode Directory [https://digital.nhs.uk/services/organisation-data-service/data-downloads/office-for-national-statistics-data], which relates current and terminated postcodes in the UK to various geographies (e.g. pre-2002 health areas, 1991 Census enumeration districts for England and Wales and 2001 and 2011 census output areas) can support the production of area-based statistics from postcoded data. Similar changes in geographies regularly occur in most countries, due to the redefining of administrative boundaries or to adjustments reflecting changes in population distribution.

### Defining the time period

Identifying the appropriate time-frame for a small-area study is another key choice that will affect the results. An area which appears to be an outlier in an annual dataset might be within the range expected when looking at multiple years. Monitoring if an excess risk persists over several time periods can indicate a signal worth investigating. The latency between a clinical outcome and exposure to a putative environmental cause needs to be considered. Whereas it is reasonable to expect respiratory complications within hours or days of exposure to high levels of air pollution,^77^ it might take several decades between exposure to a carcinogenic substance and the onset of cancer.^78^

The availability of routine data and cohorts spanning several decades has enabled the conduct of analyses of pollutant exposures and possible health effects over prolonged periods. For example, Elliott *et al.* conducted a small-area study assessing long-term mortality risks of air pollution in England and Wales and found impacts on mortality up to 16 years later.^79^ Hansell *et al.* subsequently conducted a prospective cohort study using a Census-based cohort with up to 38 years of follow-up, and concluded that air pollution exposure had long-term effects on mortality that persisted for 3 decades after exposure, and that historical air pollution exposures influenced estimates of associations between air pollution and mortality.^31^ Obtaining consistent measurements of environmental factors for the full duration of such long-term studies can present challenges, because monitoring priorities tend to shift over time, as do the accuracy and precision of measurements.

Furthermore, individuals are mobile and may change residence, particularly over long time periods. Information on mobility is often not available in routine datasets. According to the US Census Bureau, a typical resident in the USA moves on average 11 times throughout their lifetime. In England, the average number of moves over an individual’s lifetime has been estimated as eight.^80^ These changes tend to occur at key life stages such as early adulthood or during pregnancy. Tracing these changes in datasets is therefore important but can be challenging.^81^

### Data analysis

Statistical analyses of spatial data need to take account of Tobler’s first law of geography, which states that ‘everything is related to everything else, but near things are more related than distant things’.^82^ This principle is particularly useful when considering statistical smoothing techniques and spatial correlation. The SpatialEpi *R* package^83^ provides a valuable toolbox for disease mapping, cluster detection and other spatial methods.

**Figure 6:**
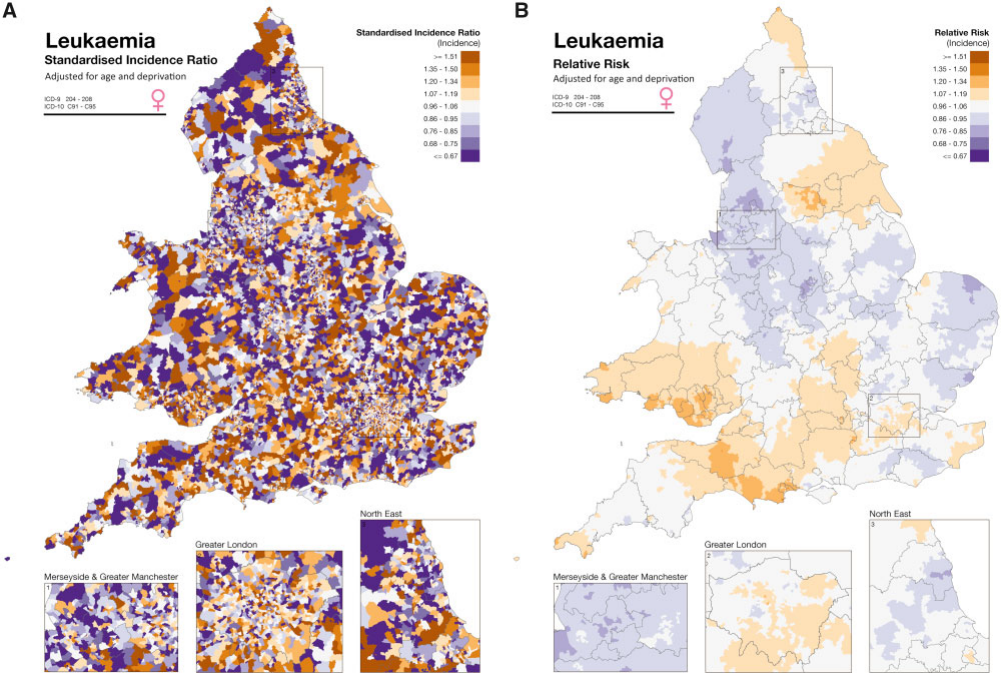
Standard incidence ratio (SIR) (A) and smoothed relative risk (B) maps of female leukaemia, adjusted for age and deprivation, in England and Wales. The two maps illustrate the impact of global and local Bayesian smoothing. Although it is hard to identify a clear pattern of disease risk in the SIR map, areas of higher and lower risk are much easier to discern with the smoothed map. Reproduced with permission from *Environment and Health Atlas of England and Wales*, as in Figure 2.

Bayesian models, in particular, have proved useful to smooth underlying risk estimates across small areas when data are sparse, providing more stable estimates of disease patterns (Figure 6).^84^ Relative risks and posterior probabilities can be derived with Bayesian smoothing approaches, with inclusion of random effects to allow for unmeasured differences between areas. Different priors are commonly used, assuming a structure of similarity either across all the areas or among neighbouring ones. The first involves ‘global smoothing’ across the whole study area, and the latter uses ‘local smoothing’ by borrowing information from neighbouring areas. A combination of the two structures can also be specified so that both ‘local’ and ‘global’ smoothing are used. Whereas such Bayesian models originally relied on computationally intensive Markov chain Monte Carlo techniques, the optimization of the Integrated Nested Laplace Approximation approach, and its integration into an *R* package R-INLA [www.r-inla.org],^85^ as well as into SAHSU’s Rapid Inquiry Facility (RIF 4.0),^86^ greatly facilitate their use.

The size of a small-area dataset can be very large when considered at regional or national scale (Table 1). Access to cloud storage can facilitate international access and collaborations, provided the necessary security protocols are in place to protect potentially identifiable data, and high-performance computing infrastructures are needed to provide the processor speed, memory and graphic interfaces to rapidly process such large datasets. Innovative software, such as DataSHIELD [http://www.datashield.ac.uk/], can enable the remote and non-disclosive analysis of sensitive research data.^79^

Traditionally, analyses have been undertaken of associations between one or several pollutants and a single or limited set of health outcomes. Recent advances in computing power and statistical methodology are facilitating multi-level analysis of complex disease aetiologies, but so far ‘big data’ health analyses have mostly focused on the analysis of -omics data. The Environment-Wide Association Study is an emerging type of environment-health analyses using a comprehensive and systematic ‘agnostic’ approach similar to that used in genome-wide association studies.^87^ Mixed-level studies^88^ allow linkage of individual-level cohort or survey data, such as biomarkers, with small-area data to better understand the associations between pollutant exposures and health, and specifically to help overcome bias and the issue of ecological confounding.^18^^,^^89^^,^^90^ Recent work on mixed-level designs has mostly focused on graphical models aiming to integrate multiple data sources at individual and small-area levels. In addition use of indices, such as the propensity score,^91^ has been proposed to summarize individual-level confounders and to impute these where missing, since surveys or cohorts may not have full spatial coverage.^92^ The estimated and imputed confounders can then be used in an ecological regression linking risk factors to health outcomes with potential to reduce bias, although care is needed in the implementation and interpretation of the models.^93^

Advances are also being made with respect to pollutant modelling using multiple data sources. The assessment of long-term exposure (e.g. to air pollutants) in small-area epidemiological studies is often based on land use regression (LUR) or dispersion models (DM). Novel methods combining LUR and DM in hybrid approaches are being developed.^94^ These advances allow modelling of previously understudied pollutants (e.g. ultrafine particles, oxidative potential of particulate matter) and back and forward extrapolation with increased temporal resolution of exposure estimates from annually to monthly or daily, while maintaining spatial granularity.^95^

In addition, spatial data mining (SDM) methods are emerging to search for patterns in large health databases, e.g. to study census and cancer mortality data in Mexico,^96^^,^^97^ or to investigate the associations between multiple exposures (such as nitrogen dioxide and fine particulate matter) and health outcomes using the HES database in England.^98^ A range of biostatistical techniques including clustering and classification approaches that can readily be scaled to large populations, are now available for such studies. Applying SDM techniques to analyse health outcomes associated with a common environmental exposure may help validate previously reported associations and identify new combinations of health effects.

Finally, advances in methods for routine surveillance of non-communicable diseases may lead to early detection of spatio-temporal signals that warrant further investigation, e.g. presence of local sources of environmental pollutants or health outcomes of extreme climatic events (temperature, flooding). One of such tools, BaySTDetect,^22^^,^^99^ includes mixture models that distinguish between areas with unusual temporal trends from those that follow a common trend.

### Protecting data privacy and confidentiality

Strict information governance and data security are essential for small-areas studies (Supplementary Material 2, available as Supplementary data at *IJE* online), since they require personal identifiable information such as residential postcode or address. Data need to be held securely with restricted access to *bona fide* staff and researchers. When releasing study results, careful attention needs to be paid to small numbers either by masking such values or by aggregating data to a higher level to avoid inadvertent disclosure of identities—including in tables and maps (e.g. rates based on small numbers of cases).

Because identifiable data from individuals are increasingly being used for a range of purposes including academic research, it is essential: (i) to inform the public about how their data are being used; (ii) to involve patients and the public where possible in the development of research projects; and (iii) to identify the best ways of communicating outcomes to the relevant audiences. Through case studies provided by UK researchers, charities and public health institutions, the Understanding Patient Data portal [http://understandingpatientdata.org.uk/] explains how and why data can be used for care and research, what is allowed and what is not, and how personal information is kept safe.

### Communicating the results

Small-area studies may reveal areas at high risk or suggest potential health effects associated with industries. Careful measured communication to the public is essential to ensure proper understanding of the size and extent of such risks, and any limitations (e.g. possible causal effects, bias and confounding) in the data and analysis. SAHSU, in collaboration with Sense about Science [http://senseaboutscience.org/], used patient and public involvement to develop the Environment and Health Atlas for England and Wales [http://www.envhealthatlas.co.uk].^100^ This atlas provides interactive maps of geographical variations for 14 health conditions, including cancers, heart disease and chronic obstructive pulmonary disease, and seven environmental agents, such as air pollutants, fungicides and herbicides, at a neighbourhood (small-area) scale. The maps were developed for the public, researchers and public health and policy professionals to better understand the geographical distribution of environmental agents and health conditions in England and Wales. Workshops with stakeholders helped in the formatting of chapters, choosing the language used to reach target audiences and the display of the maps. For example, an orange-purple palette legible by colour-blind individuals and reducing potential for misinterpretation of the risks (e.g. dangerous vs safe) was chosen instead of a red-green palette.

## Conclusions

Small-area studies are used to assess health risks in relation to environmental exposures, investigate disease clusters and carry out disease surveillance and mapping. Advantages of such studies, mainly based on routinely collected data, are their population representativeness and the lower costs and duration compared with other study designs (e.g. new cohort studied or purposely designed case-control studies). They require specialized knowledge and skills to rigorously conduct analyses, correctly interpret the results and translate them into public health policies. This is important for high-income countries, but also for low- and middle-income countries where large amounts of relevant data are being generated and where there is greater exposure to environmental toxicants. Making use of the increasing availability of large and diverse data sources for public health purposes offers great potential, although this relies on timeliness of data, rapid data linkage, necessary expertise in data management and analysis and appropriate information governance framework.

## Funding

The UK Small Area Health Statistics Unit (SAHSU) is funded by Public Health England (PHE) as part of the Medical Research Council Centre for Environment and Health, which is also supported by the Medical Research Council (MR/L01341X/1). Part of this work was supported by a Wellcome Trust Seed Award in Science to FBP (204535/Z/16/Z). 

F.B.P., M.T., A.H. and P.E. also acknowledge support from the National Institute for Health Research (NIHR) Health Protection Research Unit in Health Impact of Environmental Hazards (HPRU-2012–10141). P.E. also acknowledges support from the NIHR Imperial Biomedical Research Centre. P.E. is the Director of the UK Small Area Health Statistics Unit (SAHSU) and of the MRC-PHE Centre for Environment and Health. The views expressed are those of the authors and not necessarily those of the funders.

## Conflict of interest

None declared.

## Supplementary Material

dyaa006_Supplementary_Data
