# Supplementary material for: Small-area methods for investigation of environment and health
Source: Int J Epidemiol. 2020 Mar 17;49(2):686–99. doi: 10.1093/ije/dyaa006 (PMC7266556; doi:10.1093/ije/dyaa006)
Supplement: dyaa006_Supplementary_Data [file dyaa006_supplementary_data.docx]

# Small-area methods for investigation of environment and health

### Education Corner

Piel, F.B. ^1,2^, Fecht, D. ^1,2^, Hodgson, S.^1,2^, Blangiardo, M. ^1,2^, Toledano, M^2^, Hansell, A.L. ^1,3^ & Elliott, P.^1,2^

# Supplementary Material

Supplementary Material 1: **Comparing data from multiple databases**

In some cases, similar health or environmental data can be available from multiple sources of varying quality, completeness and time coverage. Birth data in England are collected in four different routine databases: i) the Office for National Statistics (ONS) births based on birth registrations; ii) the Hospital Episode Statistics (HES) deliveries; iii) the HES births (babies’ information); and iv) the NHS Numbers for Babies (NN4B) covering 2005-2014 based on ONS births plus pre-linked gestational age and ethnicity information from hospital systems.^41^ Ghosh *et al* ^41^ identified coding issues in certain areas and years (e.g. all births recorded as males by some hospitals) and data reporting (including local misreporting of birth weight by a factor of ten). There were important differences between the four databases, with ONS and NN4B offering the most complete and accurate record of births. More detailed clinical information was nevertheless available in HES deliveries which offers a high-quality dataset capturing the majority of English births. The choice of dataset will clearly have implications for the type and quality of studies undertaken.

Supplementary Material 2: **General Data Protection Regulation**

Across Europe, the General Data Protection Regulation (GDPR, https://www.eugdpr.org/) has recently been implemented to harmonize data privacy laws. The primary aim of this new regulation is to protect and empower all EU citizens’ data privacy, and to address growing concerns about how increasing amounts of identifiable data are collected, including through social media and internet use. Amongst the changes, the conditions for consent have been strengthened, so that consent must be clear and distinguishable from other matters and provided in an intelligible and easily accessible form, using clear and plain language. The new regulation also details specific data subject rights, such as the right to be forgotten or the right for an individual to receive the personal data concerning them that they have previously provided, and to transmit those data to another controller (data portability).
